# Supplementary material for: Pyruvate Dehydrogenase Kinase 1 inhibition mediated oxidative phosphorylation enhancement in cartilage promotes osteoarthritis progression
Source: BMC Musculoskelet Disord. 2023 Jul 20;24:597. doi: 10.1186/s12891-023-06585-6 (PMC10357736; doi:10.1186/s12891-023-06585-6)
Supplement: Supplementary file 1 — Additional file 1. [file 12891_2023_6585_MOESM1_ESM.zip › MF.Gsea.1653627316242/GOMF_PROTEIN_KINASE_ACTIVITY.html]

Details for gene set GOMF\_PROTEIN\_KINASE\_ACTIVITY[GSEA]

|  || Dataset | 55457\_collapsed\_to\_symbols.cls.cls #OA\_versus\_normal.cls.cls #OA\_versus\_normal\_repos |
| Phenotype | cls.cls#OA\_versus\_normal\_repos |
| Upregulated in class | normal |
| GeneSet | GOMF\_PROTEIN\_KINASE\_ACTIVITY |
| Enrichment Score (ES) | -0.32048815 |
| Normalized Enrichment Score (NES) | -1.6946197 |
| Nominal p-value | 0.0 |
| FDR q-value | 0.10774965 |
| FWER p-Value | 0.908 |
Table: GSEA Results Summary

  

Fig 1: Enrichment plot: GOMF\_PROTEIN\_KINASE\_ACTIVITY      
 Profile of the Running ES Score & Positions of GeneSet Members on the Rank Ordered List

  

| SYMBOL | TITLE | RANK IN GENE LIST | RANK METRIC SCORE | RUNNING ES | CORE ENRICHMENT || 1 | DYRK4 | dual specificity tyrosine phosphorylation regulated kinase 4 [Source:HGNC Symbol;Acc:HGNC:3095] | 6 | 1.217 | 0.0077 | No |
| 2 | PRKAG1 | protein kinase AMP-activated non-catalytic subunit gamma 1 [Source:HGNC Symbol;Acc:HGNC:9385] | 138 | 0.870 | 0.0031 | No |
| 3 | STK16 | serine/threonine kinase 16 [Source:HGNC Symbol;Acc:HGNC:11394] | 318 | 0.738 | -0.0063 | No |
| 4 | SRPK3 | SRSF protein kinase 3 [Source:HGNC Symbol;Acc:HGNC:11402] | 329 | 0.734 | -0.0022 | No |
| 5 | NPRL2 | "NPR2 like, GATOR1 complex subunit [Source:HGNC Symbol;Acc:HGNC:24969]" | 429 | 0.695 | -0.0054 | No |
| 6 | EPHA3 | EPH receptor A3 [Source:HGNC Symbol;Acc:HGNC:3387] | 553 | 0.641 | -0.0110 | No |
| 7 | SGK3 | serum/glucocorticoid regulated kinase family member 3 [Source:HGNC Symbol;Acc:HGNC:10812] | 609 | 0.620 | -0.0112 | No |
| 8 | CSNK1G3 | casein kinase 1 gamma 3 [Source:HGNC Symbol;Acc:HGNC:2456] | 705 | 0.592 | -0.0149 | No |
| 9 | CSF1R | colony stimulating factor 1 receptor [Source:HGNC Symbol;Acc:HGNC:2433] | 713 | 0.590 | -0.0115 | No |
| 10 | MAPK9 | mitogen-activated protein kinase 9 [Source:HGNC Symbol;Acc:HGNC:6886] | 721 | 0.588 | -0.0080 | No |
| 11 | MAPK3 | mitogen-activated protein kinase 3 [Source:HGNC Symbol;Acc:HGNC:6877] | 757 | 0.579 | -0.0069 | No |
| 12 | PRKACB | protein kinase cAMP-activated catalytic subunit beta [Source:HGNC Symbol;Acc:HGNC:9381] | 771 | 0.576 | -0.0041 | No |
| 13 | CDK7 | cyclin dependent kinase 7 [Source:HGNC Symbol;Acc:HGNC:1778] | 834 | 0.560 | -0.0053 | No |
| 14 | CPNE3 | copine 3 [Source:HGNC Symbol;Acc:HGNC:2316] | 851 | 0.554 | -0.0028 | No |
| 15 | EFNA4 | ephrin A4 [Source:HGNC Symbol;Acc:HGNC:3224] | 871 | 0.550 | -0.0007 | No |
| 16 | BTK | Bruton tyrosine kinase [Source:HGNC Symbol;Acc:HGNC:1133] | 889 | 0.545 | 0.0017 | No |
| 17 | CAMK2A | calcium/calmodulin dependent protein kinase II alpha [Source:HGNC Symbol;Acc:HGNC:1460] | 916 | 0.538 | 0.0032 | No |
| 18 | ERBB2 | erb-b2 receptor tyrosine kinase 2 [Source:HGNC Symbol;Acc:HGNC:3430] | 963 | 0.526 | 0.0031 | No |
| 19 | NEK4 | NIMA related kinase 4 [Source:HGNC Symbol;Acc:HGNC:11399] | 992 | 0.519 | 0.0043 | No |
| 20 | PDGFRL | platelet derived growth factor receptor like [Source:HGNC Symbol;Acc:HGNC:8805] | 995 | 0.519 | 0.0077 | No |
| 21 | TNNI3K | TNNI3 interacting kinase [Source:HGNC Symbol;Acc:HGNC:19661] | 1166 | 0.483 | -0.0027 | No |
| 22 | AKT1 | AKT serine/threonine kinase 1 [Source:HGNC Symbol;Acc:HGNC:391] | 1192 | 0.478 | -0.0015 | No |
| 23 | ILK | integrin linked kinase [Source:HGNC Symbol;Acc:HGNC:6040] | 1286 | 0.460 | -0.0059 | No |
| 24 | CDK4 | cyclin dependent kinase 4 [Source:HGNC Symbol;Acc:HGNC:1773] | 1375 | 0.447 | -0.0099 | No |
| 25 | MAP2K5 | mitogen-activated protein kinase kinase 5 [Source:HGNC Symbol;Acc:HGNC:6845] | 1391 | 0.444 | -0.0082 | No |
| 26 | AURKB | aurora kinase B [Source:HGNC Symbol;Acc:HGNC:11390] | 1407 | 0.442 | -0.0064 | No |
| 27 | PIK3R4 | phosphoinositide-3-kinase regulatory subunit 4 [Source:HGNC Symbol;Acc:HGNC:8982] | 1424 | 0.439 | -0.0047 | No |
| 28 | NME2 | NME/NM23 nucleoside diphosphate kinase 2 [Source:HGNC Symbol;Acc:HGNC:7850] | 1428 | 0.439 | -0.0020 | No |
| 29 | SOSTDC1 | sclerostin domain containing 1 [Source:HGNC Symbol;Acc:HGNC:21748] | 1437 | 0.437 | 0.0004 | No |
| 30 | EIF2AK1 | eukaryotic translation initiation factor 2 alpha kinase 1 [Source:HGNC Symbol;Acc:HGNC:24921] | 1442 | 0.437 | 0.0030 | No |
| 31 | HTATIP2 | HIV-1 Tat interactive protein 2 [Source:HGNC Symbol;Acc:HGNC:16637] | 1456 | 0.435 | 0.0049 | No |
| 32 | CDK14 | cyclin dependent kinase 14 [Source:HGNC Symbol;Acc:HGNC:8883] | 1549 | 0.420 | 0.0003 | No |
| 33 | ADCK2 | aarF domain containing kinase 2 [Source:HGNC Symbol;Acc:HGNC:19039] | 1631 | 0.409 | -0.0034 | No |
| 34 | FRK | fyn related Src family tyrosine kinase [Source:HGNC Symbol;Acc:HGNC:3955] | 1680 | 0.402 | -0.0046 | No |
| 35 | MAP3K7 | mitogen-activated protein kinase kinase kinase 7 [Source:HGNC Symbol;Acc:HGNC:6859] | 1701 | 0.399 | -0.0035 | No |
| 36 | EPHA7 | EPH receptor A7 [Source:HGNC Symbol;Acc:HGNC:3390] | 1770 | 0.388 | -0.0063 | No |
| 37 | PDK2 | pyruvate dehydrogenase kinase 2 [Source:HGNC Symbol;Acc:HGNC:8810] | 1772 | 0.388 | -0.0038 | No |
| 38 | CSNK2B | casein kinase 2 beta [Source:HGNC Symbol;Acc:HGNC:2460] | 1788 | 0.385 | -0.0024 | No |
| 39 | FASTK | Fas activated serine/threonine kinase [Source:HGNC Symbol;Acc:HGNC:24676] | 1834 | 0.380 | -0.0034 | No |
| 40 | LTBP1 | latent transforming growth factor beta binding protein 1 [Source:HGNC Symbol;Acc:HGNC:6714] | 1899 | 0.373 | -0.0061 | No |
| 41 | TSSK2 | testis specific serine kinase 2 [Source:HGNC Symbol;Acc:HGNC:11401] | 1968 | 0.364 | -0.0091 | No |
| 42 | CDK16 | cyclin dependent kinase 16 [Source:HGNC Symbol;Acc:HGNC:8749] | 1975 | 0.363 | -0.0071 | No |
| 43 | ROR1 | receptor tyrosine kinase like orphan receptor 1 [Source:HGNC Symbol;Acc:HGNC:10256] | 1990 | 0.362 | -0.0058 | No |
| 44 | MYO3A | myosin IIIA [Source:HGNC Symbol;Acc:HGNC:7601] | 2032 | 0.358 | -0.0067 | No |
| 45 | PINK1 | PTEN induced kinase 1 [Source:HGNC Symbol;Acc:HGNC:14581] | 2097 | 0.350 | -0.0095 | No |
| 46 | KIT | "KIT proto-oncogene, receptor tyrosine kinase [Source:HGNC Symbol;Acc:HGNC:6342]" | 2117 | 0.348 | -0.0086 | No |
| 47 | PDK1 | pyruvate dehydrogenase kinase 1 [Source:HGNC Symbol;Acc:HGNC:8809] | 2124 | 0.347 | -0.0068 | No |
| 48 | GRK6 | G protein-coupled receptor kinase 6 [Source:HGNC Symbol;Acc:HGNC:4545] | 2130 | 0.347 | -0.0048 | No |
| 49 | TNIK | TRAF2 and NCK interacting kinase [Source:HGNC Symbol;Acc:HGNC:30765] | 2133 | 0.346 | -0.0026 | No |
| 50 | CILK1 | ciliogenesis associated kinase 1 [Source:HGNC Symbol;Acc:HGNC:21219] | 2135 | 0.346 | -0.0004 | No |
| 51 | STK25 | serine/threonine kinase 25 [Source:HGNC Symbol;Acc:HGNC:11404] | 2152 | 0.344 | 0.0007 | No |
| 52 | MAP2K4 | mitogen-activated protein kinase kinase 4 [Source:HGNC Symbol;Acc:HGNC:6844] | 2214 | 0.337 | -0.0020 | No |
| 53 | NRP2 | neuropilin 2 [Source:HGNC Symbol;Acc:HGNC:8005] | 2222 | 0.336 | -0.0003 | No |
| 54 | PRKCD | protein kinase C delta [Source:HGNC Symbol;Acc:HGNC:9399] | 2237 | 0.335 | 0.0009 | No |
| 55 | LCK | "LCK proto-oncogene, Src family tyrosine kinase [Source:HGNC Symbol;Acc:HGNC:6524]" | 2274 | 0.329 | 0.0002 | No |
| 56 | PTK6 | protein tyrosine kinase 6 [Source:HGNC Symbol;Acc:HGNC:9617] | 2275 | 0.329 | 0.0024 | No |
| 57 | CASK | calcium/calmodulin dependent serine protein kinase [Source:HGNC Symbol;Acc:HGNC:1497] | 2316 | 0.324 | 0.0014 | No |
| 58 | TRIB2 | tribbles pseudokinase 2 [Source:HGNC Symbol;Acc:HGNC:30809] | 2332 | 0.323 | 0.0024 | No |
| 59 | MAPK14 | mitogen-activated protein kinase 14 [Source:HGNC Symbol;Acc:HGNC:6876] | 2352 | 0.320 | 0.0030 | No |
| 60 | BUB1 | BUB1 mitotic checkpoint serine/threonine kinase [Source:HGNC Symbol;Acc:HGNC:1148] | 2388 | 0.315 | 0.0023 | No |
| 61 | ROR2 | receptor tyrosine kinase like orphan receptor 2 [Source:HGNC Symbol;Acc:HGNC:10257] | 2448 | 0.308 | -0.0003 | No |
| 62 | TTN | titin [Source:HGNC Symbol;Acc:HGNC:12403] | 2492 | 0.303 | -0.0018 | No |
| 63 | CAMKK2 | calcium/calmodulin dependent protein kinase kinase 2 [Source:HGNC Symbol;Acc:HGNC:1470] | 2497 | 0.303 | -0.0000 | No |
| 64 | CHEK2 | checkpoint kinase 2 [Source:HGNC Symbol;Acc:HGNC:16627] | 2533 | 0.299 | -0.0008 | No |
| 65 | PKN1 | protein kinase N1 [Source:HGNC Symbol;Acc:HGNC:9405] | 2540 | 0.298 | 0.0007 | No |
| 66 | MAP4K2 | mitogen-activated protein kinase kinase kinase kinase 2 [Source:HGNC Symbol;Acc:HGNC:6864] | 2543 | 0.298 | 0.0026 | No |
| 67 | MAPK13 | mitogen-activated protein kinase 13 [Source:HGNC Symbol;Acc:HGNC:6875] | 2544 | 0.298 | 0.0046 | No |
| 68 | BLK | "BLK proto-oncogene, Src family tyrosine kinase [Source:HGNC Symbol;Acc:HGNC:1057]" | 2692 | 0.280 | -0.0054 | No |
| 69 | BMPR1A | bone morphogenetic protein receptor type 1A [Source:HGNC Symbol;Acc:HGNC:1076] | 2745 | 0.275 | -0.0077 | No |
| 70 | PRKCZ | protein kinase C zeta [Source:HGNC Symbol;Acc:HGNC:9412] | 2760 | 0.274 | -0.0069 | No |
| 71 | ULK2 | unc-51 like autophagy activating kinase 2 [Source:HGNC Symbol;Acc:HGNC:13480] | 2796 | 0.270 | -0.0079 | No |
| 72 | FGFR3 | fibroblast growth factor receptor 3 [Source:HGNC Symbol;Acc:HGNC:3690] | 2812 | 0.269 | -0.0073 | No |
| 73 | GUCY2D | "guanylate cyclase 2D, retinal [Source:HGNC Symbol;Acc:HGNC:4689]" | 2832 | 0.267 | -0.0071 | No |
| 74 | CAMK2G | calcium/calmodulin dependent protein kinase II gamma [Source:HGNC Symbol;Acc:HGNC:1463] | 2900 | 0.261 | -0.0107 | No |
| 75 | PDGFRA | platelet derived growth factor receptor alpha [Source:HGNC Symbol;Acc:HGNC:8803] | 2943 | 0.256 | -0.0123 | No |
| 76 | TXK | TXK tyrosine kinase [Source:HGNC Symbol;Acc:HGNC:12434] | 2958 | 0.254 | -0.0117 | No |
| 77 | GUCY2C | guanylate cyclase 2C [Source:HGNC Symbol;Acc:HGNC:4688] | 2982 | 0.252 | -0.0119 | No |
| 78 | NTRK1 | neurotrophic receptor tyrosine kinase 1 [Source:HGNC Symbol;Acc:HGNC:8031] | 3015 | 0.248 | -0.0128 | No |
| 79 | DDR1 | discoidin domain receptor tyrosine kinase 1 [Source:HGNC Symbol;Acc:HGNC:2730] | 3043 | 0.246 | -0.0133 | No |
| 80 | GRK4 | G protein-coupled receptor kinase 4 [Source:HGNC Symbol;Acc:HGNC:4543] | 3127 | 0.237 | -0.0184 | No |
| 81 | CHEK1 | checkpoint kinase 1 [Source:HGNC Symbol;Acc:HGNC:1925] | 3173 | 0.233 | -0.0204 | No |
| 82 | STRADA | STE20 related adaptor alpha [Source:HGNC Symbol;Acc:HGNC:30172] | 3210 | 0.229 | -0.0218 | No |
| 83 | MAP4K1 | mitogen-activated protein kinase kinase kinase kinase 1 [Source:HGNC Symbol;Acc:HGNC:6863] | 3328 | 0.219 | -0.0297 | No |
| 84 | RPS6KA1 | ribosomal protein S6 kinase A1 [Source:HGNC Symbol;Acc:HGNC:10430] | 3389 | 0.213 | -0.0331 | No |
| 85 | CDK5 | cyclin dependent kinase 5 [Source:HGNC Symbol;Acc:HGNC:1774] | 3400 | 0.211 | -0.0325 | No |
| 86 | NEK9 | NIMA related kinase 9 [Source:HGNC Symbol;Acc:HGNC:18591] | 3444 | 0.207 | -0.0345 | No |
| 87 | MAPKAPK3 | MAPK activated protein kinase 3 [Source:HGNC Symbol;Acc:HGNC:6888] | 3483 | 0.204 | -0.0362 | No |
| 88 | TRIM28 | tripartite motif containing 28 [Source:HGNC Symbol;Acc:HGNC:16384] | 3526 | 0.200 | -0.0382 | No |
| 89 | PLK1 | polo like kinase 1 [Source:HGNC Symbol;Acc:HGNC:9077] | 3556 | 0.198 | -0.0392 | No |
| 90 | STK19 | serine/threonine kinase 19 [Source:HGNC Symbol;Acc:HGNC:11398] | 3585 | 0.195 | -0.0402 | No |
| 91 | PASK | PAS domain containing serine/threonine kinase [Source:HGNC Symbol;Acc:HGNC:17270] | 3604 | 0.193 | -0.0403 | No |
| 92 | RIOK2 | RIO kinase 2 [Source:HGNC Symbol;Acc:HGNC:18999] | 3615 | 0.192 | -0.0398 | No |
| 93 | PIM2 | "Pim-2 proto-oncogene, serine/threonine kinase [Source:HGNC Symbol;Acc:HGNC:8987]" | 3623 | 0.192 | -0.0391 | No |
| 94 | PRKCQ | protein kinase C theta [Source:HGNC Symbol;Acc:HGNC:9410] | 3658 | 0.189 | -0.0405 | No |
| 95 | CAMK4 | calcium/calmodulin dependent protein kinase IV [Source:HGNC Symbol;Acc:HGNC:1464] | 3664 | 0.189 | -0.0397 | No |
| 96 | EPHB2 | EPH receptor B2 [Source:HGNC Symbol;Acc:HGNC:3393] | 3677 | 0.188 | -0.0394 | No |
| 97 | RPS6KA4 | ribosomal protein S6 kinase A4 [Source:HGNC Symbol;Acc:HGNC:10433] | 3702 | 0.185 | -0.0400 | No |
| 98 | NRP1 | neuropilin 1 [Source:HGNC Symbol;Acc:HGNC:8004] | 3704 | 0.185 | -0.0389 | No |
| 99 | PRKCA | protein kinase C alpha [Source:HGNC Symbol;Acc:HGNC:9393] | 3728 | 0.183 | -0.0395 | No |
| 100 | CDK1 | cyclin dependent kinase 1 [Source:HGNC Symbol;Acc:HGNC:1722] | 3744 | 0.182 | -0.0395 | No |
| 101 | PAK1 | p21 (RAC1) activated kinase 1 [Source:HGNC Symbol;Acc:HGNC:8590] | 3861 | 0.169 | -0.0477 | No |
| 102 | ACVR1 | activin A receptor type 1 [Source:HGNC Symbol;Acc:HGNC:171] | 3898 | 0.165 | -0.0494 | No |
| 103 | HUNK | hormonally up-regulated Neu-associated kinase [Source:HGNC Symbol;Acc:HGNC:13326] | 3996 | 0.156 | -0.0562 | No |
| 104 | SYK | spleen associated tyrosine kinase [Source:HGNC Symbol;Acc:HGNC:11491] | 4084 | 0.147 | -0.0622 | No |
| 105 | EFEMP1 | EGF containing fibulin extracellular matrix protein 1 [Source:HGNC Symbol;Acc:HGNC:3218] | 4092 | 0.147 | -0.0618 | No |
| 106 | PRKD1 | protein kinase D1 [Source:HGNC Symbol;Acc:HGNC:9407] | 4123 | 0.144 | -0.0632 | No |
| 107 | PPM1D | "protein phosphatase, Mg2+/Mn2+ dependent 1D [Source:HGNC Symbol;Acc:HGNC:9277]" | 4159 | 0.142 | -0.0651 | No |
| 108 | JAK2 | Janus kinase 2 [Source:HGNC Symbol;Acc:HGNC:6192] | 4183 | 0.140 | -0.0660 | No |
| 109 | VRK1 | VRK serine/threonine kinase 1 [Source:HGNC Symbol;Acc:HGNC:12718] | 4193 | 0.139 | -0.0658 | No |
| 110 | PSKH1 | protein serine kinase H1 [Source:HGNC Symbol;Acc:HGNC:9529] | 4199 | 0.138 | -0.0652 | No |
| 111 | RIOK3 | RIO kinase 3 [Source:HGNC Symbol;Acc:HGNC:11451] | 4206 | 0.137 | -0.0648 | No |
| 112 | GRK3 | G protein-coupled receptor kinase 3 [Source:HGNC Symbol;Acc:HGNC:290] | 4208 | 0.137 | -0.0640 | No |
| 113 | VRK3 | VRK serine/threonine kinase 3 [Source:HGNC Symbol;Acc:HGNC:18996] | 4253 | 0.133 | -0.0666 | No |
| 114 | NEK11 | NIMA related kinase 11 [Source:HGNC Symbol;Acc:HGNC:18593] | 4290 | 0.129 | -0.0686 | No |
| 115 | COQ8A | coenzyme Q8A [Source:HGNC Symbol;Acc:HGNC:16812] | 4359 | 0.124 | -0.0733 | No |
| 116 | PRKAG2 | protein kinase AMP-activated non-catalytic subunit gamma 2 [Source:HGNC Symbol;Acc:HGNC:9386] | 4362 | 0.123 | -0.0726 | No |
| 117 | MAPK7 | mitogen-activated protein kinase 7 [Source:HGNC Symbol;Acc:HGNC:6880] | 4396 | 0.121 | -0.0744 | No |
| 118 | NRBP1 | nuclear receptor binding protein 1 [Source:HGNC Symbol;Acc:HGNC:7993] | 4423 | 0.119 | -0.0757 | No |
| 119 | CCND3 | cyclin D3 [Source:HGNC Symbol;Acc:HGNC:1585] | 4426 | 0.119 | -0.0751 | No |
| 120 | BUB1B | BUB1 mitotic checkpoint serine/threonine kinase B [Source:HGNC Symbol;Acc:HGNC:1149] | 4454 | 0.117 | -0.0765 | No |
| 121 | PBK | PDZ binding kinase [Source:HGNC Symbol;Acc:HGNC:18282] | 4459 | 0.117 | -0.0760 | No |
| 122 | CSNK1D | casein kinase 1 delta [Source:HGNC Symbol;Acc:HGNC:2452] | 4529 | 0.111 | -0.0808 | No |
| 123 | TNK1 | tyrosine kinase non receptor 1 [Source:HGNC Symbol;Acc:HGNC:11940] | 4614 | 0.104 | -0.0869 | No |
| 124 | TAOK3 | TAO kinase 3 [Source:HGNC Symbol;Acc:HGNC:18133] | 4633 | 0.101 | -0.0876 | No |
| 125 | FLT3 | fms related receptor tyrosine kinase 3 [Source:HGNC Symbol;Acc:HGNC:3765] | 4657 | 0.099 | -0.0888 | No |
| 126 | POMK | protein O-mannose kinase [Source:HGNC Symbol;Acc:HGNC:26267] | 4680 | 0.098 | -0.0899 | No |
| 127 | EPHB6 | EPH receptor B6 [Source:HGNC Symbol;Acc:HGNC:3396] | 4721 | 0.094 | -0.0925 | No |
| 128 | SLA | Src like adaptor [Source:HGNC Symbol;Acc:HGNC:10902] | 4786 | 0.088 | -0.0970 | No |
| 129 | RIPK2 | receptor interacting serine/threonine kinase 2 [Source:HGNC Symbol;Acc:HGNC:10020] | 4848 | 0.083 | -0.1014 | No |
| 130 | ERBB3 | erb-b2 receptor tyrosine kinase 3 [Source:HGNC Symbol;Acc:HGNC:3431] | 4863 | 0.083 | -0.1020 | No |
| 131 | RASSF2 | Ras association domain family member 2 [Source:HGNC Symbol;Acc:HGNC:9883] | 4894 | 0.080 | -0.1038 | No |
| 132 | AURKA | aurora kinase A [Source:HGNC Symbol;Acc:HGNC:11393] | 4916 | 0.079 | -0.1050 | No |
| 133 | VRK2 | VRK serine/threonine kinase 2 [Source:HGNC Symbol;Acc:HGNC:12719] | 4918 | 0.078 | -0.1045 | No |
| 134 | TESK2 | testis associated actin remodelling kinase 2 [Source:HGNC Symbol;Acc:HGNC:11732] | 4947 | 0.076 | -0.1063 | No |
| 135 | MYLK | myosin light chain kinase [Source:HGNC Symbol;Acc:HGNC:7590] | 5079 | 0.066 | -0.1164 | No |
| 136 | MERTK | "MER proto-oncogene, tyrosine kinase [Source:HGNC Symbol;Acc:HGNC:7027]" | 5085 | 0.066 | -0.1163 | No |
| 137 | NUAK2 | NUAK family kinase 2 [Source:HGNC Symbol;Acc:HGNC:29558] | 5087 | 0.065 | -0.1160 | No |
| 138 | CDC42BPA | CDC42 binding protein kinase alpha [Source:HGNC Symbol;Acc:HGNC:1737] | 5097 | 0.065 | -0.1163 | No |
| 139 | HSPB8 | heat shock protein family B (small) member 8 [Source:HGNC Symbol;Acc:HGNC:30171] | 5141 | 0.060 | -0.1193 | No |
| 140 | EIF2AK2 | eukaryotic translation initiation factor 2 alpha kinase 2 [Source:HGNC Symbol;Acc:HGNC:9437] | 5145 | 0.060 | -0.1192 | No |
| 141 | NEK7 | NIMA related kinase 7 [Source:HGNC Symbol;Acc:HGNC:13386] | 5147 | 0.060 | -0.1188 | No |
| 142 | MUSK | muscle associated receptor tyrosine kinase [Source:HGNC Symbol;Acc:HGNC:7525] | 5166 | 0.059 | -0.1199 | No |
| 143 | DYRK2 | dual specificity tyrosine phosphorylation regulated kinase 2 [Source:HGNC Symbol;Acc:HGNC:3093] | 5224 | 0.054 | -0.1241 | No |
| 144 | OBSCN | "obscurin, cytoskeletal calmodulin and titin-interacting RhoGEF [Source:HGNC Symbol;Acc:HGNC:15719]" | 5230 | 0.054 | -0.1241 | No |
| 145 | TLK1 | tousled like kinase 1 [Source:HGNC Symbol;Acc:HGNC:11841] | 5232 | 0.054 | -0.1239 | No |
| 146 | AURKC | aurora kinase C [Source:HGNC Symbol;Acc:HGNC:11391] | 5256 | 0.052 | -0.1254 | No |
| 147 | TGFBR1 | transforming growth factor beta receptor 1 [Source:HGNC Symbol;Acc:HGNC:11772] | 5280 | 0.050 | -0.1269 | No |
| 148 | MMD | monocyte to macrophage differentiation associated [Source:HGNC Symbol;Acc:HGNC:7153] | 5288 | 0.049 | -0.1271 | No |
| 149 | MYLK3 | myosin light chain kinase 3 [Source:HGNC Symbol;Acc:HGNC:29826] | 5294 | 0.048 | -0.1272 | No |
| 150 | FGFR4 | fibroblast growth factor receptor 4 [Source:HGNC Symbol;Acc:HGNC:3691] | 5351 | 0.044 | -0.1314 | No |
| 151 | NTRK2 | neurotrophic receptor tyrosine kinase 2 [Source:HGNC Symbol;Acc:HGNC:8032] | 5431 | 0.038 | -0.1375 | No |
| 152 | MATK | megakaryocyte-associated tyrosine kinase [Source:HGNC Symbol;Acc:HGNC:6906] | 5462 | 0.036 | -0.1397 | No |
| 153 | SIK3 | SIK family kinase 3 [Source:HGNC Symbol;Acc:HGNC:29165] | 5476 | 0.035 | -0.1405 | No |
| 154 | CAMK1G | calcium/calmodulin dependent protein kinase IG [Source:HGNC Symbol;Acc:HGNC:14585] | 5482 | 0.034 | -0.1407 | No |
| 155 | RPS6KB2 | ribosomal protein S6 kinase B2 [Source:HGNC Symbol;Acc:HGNC:10437] | 5496 | 0.033 | -0.1415 | No |
| 156 | MAST1 | microtubule associated serine/threonine kinase 1 [Source:HGNC Symbol;Acc:HGNC:19034] | 5559 | 0.028 | -0.1463 | No |
| 157 | MAP3K19 | mitogen-activated protein kinase kinase kinase 19 [Source:HGNC Symbol;Acc:HGNC:26249] | 5571 | 0.026 | -0.1470 | No |
| 158 | EPHB3 | EPH receptor B3 [Source:HGNC Symbol;Acc:HGNC:3394] | 5614 | 0.023 | -0.1502 | No |
| 159 | CDKL5 | cyclin dependent kinase like 5 [Source:HGNC Symbol;Acc:HGNC:11411] | 5655 | 0.019 | -0.1533 | No |
| 160 | ERN1 | endoplasmic reticulum to nucleus signaling 1 [Source:HGNC Symbol;Acc:HGNC:3449] | 5658 | 0.019 | -0.1533 | No |
| 161 | MAP3K13 | mitogen-activated protein kinase kinase kinase 13 [Source:HGNC Symbol;Acc:HGNC:6852] | 5728 | 0.013 | -0.1588 | No |
| 162 | CDK19 | cyclin dependent kinase 19 [Source:HGNC Symbol;Acc:HGNC:19338] | 5733 | 0.013 | -0.1590 | No |
| 163 | BMP2K | BMP2 inducible kinase [Source:HGNC Symbol;Acc:HGNC:18041] | 5760 | 0.011 | -0.1611 | No |
| 164 | MKNK1 | MAPK interacting serine/threonine kinase 1 [Source:HGNC Symbol;Acc:HGNC:7110] | 5830 | 0.007 | -0.1666 | No |
| 165 | STK10 | serine/threonine kinase 10 [Source:HGNC Symbol;Acc:HGNC:11388] | 5883 | 0.003 | -0.1707 | No |
| 166 | RYK | receptor like tyrosine kinase [Source:HGNC Symbol;Acc:HGNC:10481] | 5896 | 0.002 | -0.1717 | No |
| 167 | IKBKB | inhibitor of nuclear factor kappa B kinase subunit beta [Source:HGNC Symbol;Acc:HGNC:5960] | 5914 | 0.001 | -0.1731 | No |
| 168 | PRKCE | protein kinase C epsilon [Source:HGNC Symbol;Acc:HGNC:9401] | 5926 | 0.000 | -0.1739 | No |
| 169 | BMPR1B | bone morphogenetic protein receptor type 1B [Source:HGNC Symbol;Acc:HGNC:1077] | 5959 | -0.002 | -0.1765 | No |
| 170 | ACVR2A | activin A receptor type 2A [Source:HGNC Symbol;Acc:HGNC:173] | 5960 | -0.002 | -0.1765 | No |
| 171 | ITK | IL2 inducible T cell kinase [Source:HGNC Symbol;Acc:HGNC:6171] | 6008 | -0.005 | -0.1802 | No |
| 172 | PLK4 | polo like kinase 4 [Source:HGNC Symbol;Acc:HGNC:11397] | 6041 | -0.008 | -0.1828 | No |
| 173 | MAPKAPK5 | MAPK activated protein kinase 5 [Source:HGNC Symbol;Acc:HGNC:6889] | 6094 | -0.012 | -0.1869 | No |
| 174 | TYRO3 | TYRO3 protein tyrosine kinase [Source:HGNC Symbol;Acc:HGNC:12446] | 6102 | -0.013 | -0.1873 | No |
| 175 | CSNK2A2 | casein kinase 2 alpha 2 [Source:HGNC Symbol;Acc:HGNC:2459] | 6105 | -0.013 | -0.1874 | No |
| 176 | BCKDK | branched chain keto acid dehydrogenase kinase [Source:HGNC Symbol;Acc:HGNC:16902] | 6112 | -0.014 | -0.1878 | No |
| 177 | ATM | ATM serine/threonine kinase [Source:HGNC Symbol;Acc:HGNC:795] | 6179 | -0.019 | -0.1930 | No |
| 178 | TEX14 | "testis expressed 14, intercellular bridge forming factor [Source:HGNC Symbol;Acc:HGNC:11737]" | 6182 | -0.019 | -0.1930 | No |
| 179 | RAF1 | "Raf-1 proto-oncogene, serine/threonine kinase [Source:HGNC Symbol;Acc:HGNC:9829]" | 6187 | -0.019 | -0.1932 | No |
| 180 | TLK2 | tousled like kinase 2 [Source:HGNC Symbol;Acc:HGNC:11842] | 6190 | -0.020 | -0.1932 | No |
| 181 | GRK5 | G protein-coupled receptor kinase 5 [Source:HGNC Symbol;Acc:HGNC:4544] | 6209 | -0.022 | -0.1945 | No |
| 182 | BRSK2 | BR serine/threonine kinase 2 [Source:HGNC Symbol;Acc:HGNC:11405] | 6238 | -0.023 | -0.1966 | No |
| 183 | SQSTM1 | sequestosome 1 [Source:HGNC Symbol;Acc:HGNC:11280] | 6260 | -0.025 | -0.1982 | No |
| 184 | BMX | BMX non-receptor tyrosine kinase [Source:HGNC Symbol;Acc:HGNC:1079] | 6265 | -0.025 | -0.1983 | No |
| 185 | NEK1 | NIMA related kinase 1 [Source:HGNC Symbol;Acc:HGNC:7744] | 6266 | -0.025 | -0.1982 | No |
| 186 | CAD | "carbamoyl-phosphate synthetase 2, aspartate transcarbamylase, and dihydroorotase [Source:HGNC Symbol;Acc:HGNC:1424]" | 6359 | -0.032 | -0.2053 | No |
| 187 | ACVR1B | activin A receptor type 1B [Source:HGNC Symbol;Acc:HGNC:172] | 6368 | -0.033 | -0.2058 | No |
| 188 | CLK3 | CDC like kinase 3 [Source:HGNC Symbol;Acc:HGNC:2071] | 6417 | -0.037 | -0.2094 | No |
| 189 | NEK2 | NIMA related kinase 2 [Source:HGNC Symbol;Acc:HGNC:7745] | 6422 | -0.037 | -0.2094 | No |
| 190 | MELK | maternal embryonic leucine zipper kinase [Source:HGNC Symbol;Acc:HGNC:16870] | 6424 | -0.037 | -0.2093 | No |
| 191 | PHKG1 | phosphorylase kinase catalytic subunit gamma 1 [Source:HGNC Symbol;Acc:HGNC:8930] | 6451 | -0.039 | -0.2111 | No |
| 192 | PRKCB | protein kinase C beta [Source:HGNC Symbol;Acc:HGNC:9395] | 6453 | -0.039 | -0.2109 | No |
| 193 | FES | "FES proto-oncogene, tyrosine kinase [Source:HGNC Symbol;Acc:HGNC:3657]" | 6491 | -0.042 | -0.2136 | No |
| 194 | GUCY2F | "guanylate cyclase 2F, retinal [Source:HGNC Symbol;Acc:HGNC:4691]" | 6510 | -0.043 | -0.2148 | No |
| 195 | SPEG | striated muscle enriched protein kinase [Source:HGNC Symbol;Acc:HGNC:16901] | 6512 | -0.043 | -0.2146 | No |
| 196 | LYN | "LYN proto-oncogene, Src family tyrosine kinase [Source:HGNC Symbol;Acc:HGNC:6735]" | 6514 | -0.043 | -0.2144 | No |
| 197 | STK38L | serine/threonine kinase 38 like [Source:HGNC Symbol;Acc:HGNC:17848] | 6540 | -0.045 | -0.2161 | No |
| 198 | CCL5 | C-C motif chemokine ligand 5 [Source:HGNC Symbol;Acc:HGNC:10632] | 6584 | -0.048 | -0.2192 | No |
| 199 | SGK2 | serum/glucocorticoid regulated kinase 2 [Source:HGNC Symbol;Acc:HGNC:13900] | 6597 | -0.049 | -0.2198 | No |
| 200 | TTK | TTK protein kinase [Source:HGNC Symbol;Acc:HGNC:12401] | 6670 | -0.054 | -0.2253 | No |
| 201 | PRKD3 | protein kinase D3 [Source:HGNC Symbol;Acc:HGNC:9408] | 6717 | -0.057 | -0.2286 | No |
| 202 | NPR2 | natriuretic peptide receptor 2 [Source:HGNC Symbol;Acc:HGNC:7944] | 6771 | -0.061 | -0.2324 | No |
| 203 | HCK | "HCK proto-oncogene, Src family tyrosine kinase [Source:HGNC Symbol;Acc:HGNC:4840]" | 6788 | -0.062 | -0.2333 | No |
| 204 | ERBB4 | erb-b2 receptor tyrosine kinase 4 [Source:HGNC Symbol;Acc:HGNC:3432] | 6830 | -0.065 | -0.2362 | No |
| 205 | MAPK1 | mitogen-activated protein kinase 1 [Source:HGNC Symbol;Acc:HGNC:6871] | 6853 | -0.068 | -0.2375 | No |
| 206 | TGFBR3 | transforming growth factor beta receptor 3 [Source:HGNC Symbol;Acc:HGNC:11774] | 6868 | -0.069 | -0.2381 | No |
| 207 | PDGFRB | platelet derived growth factor receptor beta [Source:HGNC Symbol;Acc:HGNC:8804] | 6914 | -0.073 | -0.2413 | No |
| 208 | LTBP4 | latent transforming growth factor beta binding protein 4 [Source:HGNC Symbol;Acc:HGNC:6717] | 6933 | -0.074 | -0.2422 | No |
| 209 | SRC | "SRC proto-oncogene, non-receptor tyrosine kinase [Source:HGNC Symbol;Acc:HGNC:11283]" | 6940 | -0.075 | -0.2422 | No |
| 210 | ZAP70 | zeta chain of T cell receptor associated protein kinase 70 [Source:HGNC Symbol;Acc:HGNC:12858] | 6962 | -0.077 | -0.2434 | No |
| 211 | MAP3K2 | mitogen-activated protein kinase kinase kinase 2 [Source:HGNC Symbol;Acc:HGNC:6854] | 6976 | -0.078 | -0.2439 | No |
| 212 | JAK1 | Janus kinase 1 [Source:HGNC Symbol;Acc:HGNC:6190] | 6995 | -0.079 | -0.2448 | No |
| 213 | PRKD2 | protein kinase D2 [Source:HGNC Symbol;Acc:HGNC:17293] | 7011 | -0.081 | -0.2455 | No |
| 214 | STK17A | serine/threonine kinase 17a [Source:HGNC Symbol;Acc:HGNC:11395] | 7016 | -0.081 | -0.2452 | No |
| 215 | MAP3K1 | mitogen-activated protein kinase kinase kinase 1 [Source:HGNC Symbol;Acc:HGNC:6848] | 7066 | -0.085 | -0.2486 | No |
| 216 | RPS6KA3 | ribosomal protein S6 kinase A3 [Source:HGNC Symbol;Acc:HGNC:10432] | 7092 | -0.087 | -0.2500 | No |
| 217 | PRKCH | protein kinase C eta [Source:HGNC Symbol;Acc:HGNC:9403] | 7141 | -0.092 | -0.2533 | No |
| 218 | AVP | arginine vasopressin [Source:HGNC Symbol;Acc:HGNC:894] | 7201 | -0.096 | -0.2574 | No |
| 219 | ENG | endoglin [Source:HGNC Symbol;Acc:HGNC:3349] | 7213 | -0.097 | -0.2576 | No |
| 220 | TWF1 | twinfilin actin binding protein 1 [Source:HGNC Symbol;Acc:HGNC:9620] | 7232 | -0.098 | -0.2584 | No |
| 221 | MAP2K6 | mitogen-activated protein kinase kinase 6 [Source:HGNC Symbol;Acc:HGNC:6846] | 7246 | -0.099 | -0.2588 | No |
| 222 | PTK2 | protein tyrosine kinase 2 [Source:HGNC Symbol;Acc:HGNC:9611] | 7315 | -0.103 | -0.2635 | No |
| 223 | MET | "MET proto-oncogene, receptor tyrosine kinase [Source:HGNC Symbol;Acc:HGNC:7029]" | 7322 | -0.104 | -0.2633 | No |
| 224 | NLK | nemo like kinase [Source:HGNC Symbol;Acc:HGNC:29858] | 7336 | -0.105 | -0.2636 | No |
| 225 | PEAK1 | pseudopodium enriched atypical kinase 1 [Source:HGNC Symbol;Acc:HGNC:29431] | 7362 | -0.107 | -0.2649 | No |
| 226 | FGFR2 | fibroblast growth factor receptor 2 [Source:HGNC Symbol;Acc:HGNC:3689] | 7374 | -0.108 | -0.2651 | No |
| 227 | CAMKV | CaM kinase like vesicle associated [Source:HGNC Symbol;Acc:HGNC:28788] | 7454 | -0.115 | -0.2707 | No |
| 228 | MINK1 | misshapen like kinase 1 [Source:HGNC Symbol;Acc:HGNC:17565] | 7488 | -0.117 | -0.2725 | No |
| 229 | RIPK4 | receptor interacting serine/threonine kinase 4 [Source:HGNC Symbol;Acc:HGNC:496] | 7505 | -0.119 | -0.2730 | No |
| 230 | CCND1 | cyclin D1 [Source:HGNC Symbol;Acc:HGNC:1582] | 7512 | -0.119 | -0.2727 | No |
| 231 | CDKL3 | cyclin dependent kinase like 3 [Source:HGNC Symbol;Acc:HGNC:15483] | 7527 | -0.121 | -0.2730 | No |
| 232 | PAK5 | p21 (RAC1) activated kinase 5 [Source:HGNC Symbol;Acc:HGNC:15916] | 7558 | -0.124 | -0.2746 | No |
| 233 | ERN2 | endoplasmic reticulum to nucleus signaling 2 [Source:HGNC Symbol;Acc:HGNC:16942] | 7565 | -0.124 | -0.2742 | No |
| 234 | TESK1 | testis associated actin remodelling kinase 1 [Source:HGNC Symbol;Acc:HGNC:11731] | 7633 | -0.129 | -0.2787 | No |
| 235 | PDK3 | pyruvate dehydrogenase kinase 3 [Source:HGNC Symbol;Acc:HGNC:8811] | 7661 | -0.132 | -0.2800 | No |
| 236 | DYRK1A | dual specificity tyrosine phosphorylation regulated kinase 1A [Source:HGNC Symbol;Acc:HGNC:3091] | 7667 | -0.132 | -0.2795 | No |
| 237 | ROS1 | "ROS proto-oncogene 1, receptor tyrosine kinase [Source:HGNC Symbol;Acc:HGNC:10261]" | 7673 | -0.133 | -0.2790 | No |
| 238 | PTK7 | protein tyrosine kinase 7 (inactive) [Source:HGNC Symbol;Acc:HGNC:9618] | 7725 | -0.136 | -0.2822 | No |
| 239 | PIK3CA | "phosphatidylinositol-4,5-bisphosphate 3-kinase catalytic subunit alpha [Source:HGNC Symbol;Acc:HGNC:8975]" | 7733 | -0.136 | -0.2819 | No |
| 240 | PAK2 | p21 (RAC1) activated kinase 2 [Source:HGNC Symbol;Acc:HGNC:8591] | 7762 | -0.139 | -0.2832 | No |
| 241 | EPHA1 | EPH receptor A1 [Source:HGNC Symbol;Acc:HGNC:3385] | 7771 | -0.139 | -0.2829 | No |
| 242 | CAMK1 | calcium/calmodulin dependent protein kinase I [Source:HGNC Symbol;Acc:HGNC:1459] | 7795 | -0.141 | -0.2838 | No |
| 243 | IGF2R | insulin like growth factor 2 receptor [Source:HGNC Symbol;Acc:HGNC:5467] | 7829 | -0.143 | -0.2855 | No |
| 244 | RPS6KC1 | ribosomal protein S6 kinase C1 [Source:HGNC Symbol;Acc:HGNC:10439] | 7856 | -0.145 | -0.2866 | No |
| 245 | DCLK1 | doublecortin like kinase 1 [Source:HGNC Symbol;Acc:HGNC:2700] | 7861 | -0.145 | -0.2859 | No |
| 246 | PHKA1 | phosphorylase kinase regulatory subunit alpha 1 [Source:HGNC Symbol;Acc:HGNC:8925] | 7896 | -0.147 | -0.2877 | No |
| 247 | FGFR1 | fibroblast growth factor receptor 1 [Source:HGNC Symbol;Acc:HGNC:3688] | 7905 | -0.148 | -0.2873 | No |
| 248 | CDC42BPB | CDC42 binding protein kinase beta [Source:HGNC Symbol;Acc:HGNC:1738] | 7943 | -0.151 | -0.2893 | No |
| 249 | MAP4K5 | mitogen-activated protein kinase kinase kinase kinase 5 [Source:HGNC Symbol;Acc:HGNC:6867] | 7950 | -0.151 | -0.2887 | No |
| 250 | PIK3CG | "phosphatidylinositol-4,5-bisphosphate 3-kinase catalytic subunit gamma [Source:HGNC Symbol;Acc:HGNC:8978]" | 7953 | -0.152 | -0.2879 | No |
| 251 | AKT3 | AKT serine/threonine kinase 3 [Source:HGNC Symbol;Acc:HGNC:393] | 8008 | -0.156 | -0.2912 | No |
| 252 | RPS6KA6 | ribosomal protein S6 kinase A6 [Source:HGNC Symbol;Acc:HGNC:10435] | 8071 | -0.161 | -0.2951 | No |
| 253 | ARAF | "A-Raf proto-oncogene, serine/threonine kinase [Source:HGNC Symbol;Acc:HGNC:646]" | 8092 | -0.163 | -0.2956 | No |
| 254 | MAP3K14 | mitogen-activated protein kinase kinase kinase 14 [Source:HGNC Symbol;Acc:HGNC:6853] | 8100 | -0.163 | -0.2950 | No |
| 255 | PIK3C3 | phosphatidylinositol 3-kinase catalytic subunit type 3 [Source:HGNC Symbol;Acc:HGNC:8974] | 8141 | -0.166 | -0.2971 | No |
| 256 | MAPK12 | mitogen-activated protein kinase 12 [Source:HGNC Symbol;Acc:HGNC:6874] | 8161 | -0.168 | -0.2975 | No |
| 257 | BRAF | "B-Raf proto-oncogene, serine/threonine kinase [Source:HGNC Symbol;Acc:HGNC:1097]" | 8171 | -0.169 | -0.2971 | No |
| 258 | CDK17 | cyclin dependent kinase 17 [Source:HGNC Symbol;Acc:HGNC:8750] | 8175 | -0.169 | -0.2962 | No |
| 259 | EFNA3 | ephrin A3 [Source:HGNC Symbol;Acc:HGNC:3223] | 8194 | -0.171 | -0.2965 | No |
| 260 | TGFBR2 | transforming growth factor beta receptor 2 [Source:HGNC Symbol;Acc:HGNC:11773] | 8241 | -0.174 | -0.2990 | No |
| 261 | FGR | "FGR proto-oncogene, Src family tyrosine kinase [Source:HGNC Symbol;Acc:HGNC:3697]" | 8248 | -0.175 | -0.2983 | No |
| 262 | PAK3 | p21 (RAC1) activated kinase 3 [Source:HGNC Symbol;Acc:HGNC:8592] | 8266 | -0.176 | -0.2985 | No |
| 263 | PHKA2 | phosphorylase kinase regulatory subunit alpha 2 [Source:HGNC Symbol;Acc:HGNC:8926] | 8333 | -0.181 | -0.3026 | No |
| 264 | EPHB1 | EPH receptor B1 [Source:HGNC Symbol;Acc:HGNC:3392] | 8342 | -0.181 | -0.3020 | No |
| 265 | DYRK1B | dual specificity tyrosine phosphorylation regulated kinase 1B [Source:HGNC Symbol;Acc:HGNC:3092] | 8346 | -0.182 | -0.3010 | No |
| 266 | CDK6 | cyclin dependent kinase 6 [Source:HGNC Symbol;Acc:HGNC:1777] | 8393 | -0.185 | -0.3035 | No |
| 267 | RPS6KA2 | ribosomal protein S6 kinase A2 [Source:HGNC Symbol;Acc:HGNC:10431] | 8397 | -0.185 | -0.3025 | No |
| 268 | GRK2 | G protein-coupled receptor kinase 2 [Source:HGNC Symbol;Acc:HGNC:289] | 8470 | -0.191 | -0.3070 | No |
| 269 | AXL | AXL receptor tyrosine kinase [Source:HGNC Symbol;Acc:HGNC:905] | 8565 | -0.199 | -0.3132 | No |
| 270 | PRKCG | protein kinase C gamma [Source:HGNC Symbol;Acc:HGNC:9402] | 8582 | -0.200 | -0.3131 | No |
| 271 | DAPK1 | death associated protein kinase 1 [Source:HGNC Symbol;Acc:HGNC:2674] | 8588 | -0.200 | -0.3122 | No |
| 272 | TEK | TEK receptor tyrosine kinase [Source:HGNC Symbol;Acc:HGNC:11724] | 8623 | -0.203 | -0.3136 | No |
| 273 | MAP3K4 | mitogen-activated protein kinase kinase kinase 4 [Source:HGNC Symbol;Acc:HGNC:6856] | 8637 | -0.203 | -0.3132 | No |
| 274 | MAST4 | microtubule associated serine/threonine kinase family member 4 [Source:HGNC Symbol;Acc:HGNC:19037] | 8667 | -0.206 | -0.3142 | No |
| 275 | PRKAB1 | protein kinase AMP-activated non-catalytic subunit beta 1 [Source:HGNC Symbol;Acc:HGNC:9378] | 8708 | -0.210 | -0.3160 | No |
| 276 | MAPK6 | mitogen-activated protein kinase 6 [Source:HGNC Symbol;Acc:HGNC:6879] | 8710 | -0.210 | -0.3146 | No |
| 277 | DSTYK | dual serine/threonine and tyrosine protein kinase [Source:HGNC Symbol;Acc:HGNC:29043] | 8749 | -0.212 | -0.3163 | No |
| 278 | CLK4 | CDC like kinase 4 [Source:HGNC Symbol;Acc:HGNC:13659] | 8760 | -0.213 | -0.3156 | No |
| 279 | NEK3 | NIMA related kinase 3 [Source:HGNC Symbol;Acc:HGNC:7746] | 8762 | -0.213 | -0.3143 | No |
| 280 | DCAF1 | DDB1 and CUL4 associated factor 1 [Source:HGNC Symbol;Acc:HGNC:30911] | 8765 | -0.213 | -0.3130 | No |
| 281 | KSR1 | kinase suppressor of ras 1 [Source:HGNC Symbol;Acc:HGNC:6465] | 8774 | -0.214 | -0.3122 | No |
| 282 | CAMK2B | calcium/calmodulin dependent protein kinase II beta [Source:HGNC Symbol;Acc:HGNC:1461] | 8782 | -0.215 | -0.3113 | No |
| 283 | DAPK2 | death associated protein kinase 2 [Source:HGNC Symbol;Acc:HGNC:2675] | 8818 | -0.219 | -0.3126 | No |
| 284 | TEC | tec protein tyrosine kinase [Source:HGNC Symbol;Acc:HGNC:11719] | 8839 | -0.220 | -0.3127 | No |
| 285 | PRKAA2 | protein kinase AMP-activated catalytic subunit alpha 2 [Source:HGNC Symbol;Acc:HGNC:9377] | 8864 | -0.222 | -0.3132 | No |
| 286 | LRRK1 | leucine rich repeat kinase 1 [Source:HGNC Symbol;Acc:HGNC:18608] | 8881 | -0.223 | -0.3129 | No |
| 287 | OXSR1 | oxidative stress responsive kinase 1 [Source:HGNC Symbol;Acc:HGNC:8508] | 8897 | -0.224 | -0.3126 | No |
| 288 | WEE1 | WEE1 G2 checkpoint kinase [Source:HGNC Symbol;Acc:HGNC:12761] | 8937 | -0.227 | -0.3142 | No |
| 289 | TNK2 | tyrosine kinase non receptor 2 [Source:HGNC Symbol;Acc:HGNC:19297] | 8955 | -0.228 | -0.3141 | No |
| 290 | MAPK8 | mitogen-activated protein kinase 8 [Source:HGNC Symbol;Acc:HGNC:6881] | 8956 | -0.228 | -0.3125 | No |
| 291 | CSK | C-terminal Src kinase [Source:HGNC Symbol;Acc:HGNC:2444] | 9056 | -0.236 | -0.3189 | Yes |
| 292 | PAK6 | p21 (RAC1) activated kinase 6 [Source:HGNC Symbol;Acc:HGNC:16061] | 9068 | -0.237 | -0.3182 | Yes |
| 293 | ABL1 | "ABL proto-oncogene 1, non-receptor tyrosine kinase [Source:HGNC Symbol;Acc:HGNC:76]" | 9081 | -0.238 | -0.3175 | Yes |
| 294 | PCK1 | phosphoenolpyruvate carboxykinase 1 [Source:HGNC Symbol;Acc:HGNC:8724] | 9100 | -0.239 | -0.3174 | Yes |
| 295 | PRKG2 | protein kinase cGMP-dependent 2 [Source:HGNC Symbol;Acc:HGNC:9416] | 9126 | -0.242 | -0.3177 | Yes |
| 296 | MOS | "MOS proto-oncogene, serine/threonine kinase [Source:HGNC Symbol;Acc:HGNC:7199]" | 9138 | -0.243 | -0.3170 | Yes |
| 297 | TSSK1B | testis specific serine kinase 1B [Source:HGNC Symbol;Acc:HGNC:14968] | 9178 | -0.246 | -0.3185 | Yes |
| 298 | CIITA | class II major histocompatibility complex transactivator [Source:HGNC Symbol;Acc:HGNC:7067] | 9184 | -0.246 | -0.3172 | Yes |
| 299 | NUAK1 | NUAK family kinase 1 [Source:HGNC Symbol;Acc:HGNC:14311] | 9190 | -0.247 | -0.3159 | Yes |
| 300 | STK39 | serine/threonine kinase 39 [Source:HGNC Symbol;Acc:HGNC:17717] | 9206 | -0.248 | -0.3155 | Yes |
| 301 | PRPF4B | pre-mRNA processing factor 4B [Source:HGNC Symbol;Acc:HGNC:17346] | 9210 | -0.249 | -0.3140 | Yes |
| 302 | CDK12 | cyclin dependent kinase 12 [Source:HGNC Symbol;Acc:HGNC:24224] | 9225 | -0.250 | -0.3134 | Yes |
| 303 | MAST3 | microtubule associated serine/threonine kinase 3 [Source:HGNC Symbol;Acc:HGNC:19036] | 9235 | -0.251 | -0.3125 | Yes |
| 304 | STK32B | serine/threonine kinase 32B [Source:HGNC Symbol;Acc:HGNC:14217] | 9258 | -0.252 | -0.3125 | Yes |
| 305 | RET | ret proto-oncogene [Source:HGNC Symbol;Acc:HGNC:9967] | 9297 | -0.256 | -0.3139 | Yes |
| 306 | MAP3K11 | mitogen-activated protein kinase kinase kinase 11 [Source:HGNC Symbol;Acc:HGNC:6850] | 9331 | -0.258 | -0.3148 | Yes |
| 307 | CIT | citron rho-interacting serine/threonine kinase [Source:HGNC Symbol;Acc:HGNC:1985] | 9356 | -0.260 | -0.3150 | Yes |
| 308 | SLK | STE20 like kinase [Source:HGNC Symbol;Acc:HGNC:11088] | 9362 | -0.261 | -0.3136 | Yes |
| 309 | IRAK1 | interleukin 1 receptor associated kinase 1 [Source:HGNC Symbol;Acc:HGNC:6112] | 9377 | -0.262 | -0.3129 | Yes |
| 310 | CSNK1G1 | casein kinase 1 gamma 1 [Source:HGNC Symbol;Acc:HGNC:2454] | 9378 | -0.262 | -0.3112 | Yes |
| 311 | RPS6KA5 | ribosomal protein S6 kinase A5 [Source:HGNC Symbol;Acc:HGNC:10434] | 9406 | -0.265 | -0.3116 | Yes |
| 312 | CDKL2 | cyclin dependent kinase like 2 [Source:HGNC Symbol;Acc:HGNC:1782] | 9453 | -0.269 | -0.3134 | Yes |
| 313 | PLK2 | polo like kinase 2 [Source:HGNC Symbol;Acc:HGNC:19699] | 9482 | -0.273 | -0.3139 | Yes |
| 314 | CDK8 | cyclin dependent kinase 8 [Source:HGNC Symbol;Acc:HGNC:1779] | 9513 | -0.275 | -0.3144 | Yes |
| 315 | MAP4K3 | mitogen-activated protein kinase kinase kinase kinase 3 [Source:HGNC Symbol;Acc:HGNC:6865] | 9520 | -0.276 | -0.3130 | Yes |
| 316 | EPHA5 | EPH receptor A5 [Source:HGNC Symbol;Acc:HGNC:3389] | 9541 | -0.277 | -0.3128 | Yes |
| 317 | TIE1 | tyrosine kinase with immunoglobulin like and EGF like domains 1 [Source:HGNC Symbol;Acc:HGNC:11809] | 9548 | -0.278 | -0.3114 | Yes |
| 318 | CDK18 | cyclin dependent kinase 18 [Source:HGNC Symbol;Acc:HGNC:8751] | 9580 | -0.281 | -0.3120 | Yes |
| 319 | MARK4 | microtubule affinity regulating kinase 4 [Source:HGNC Symbol;Acc:HGNC:13538] | 9590 | -0.282 | -0.3108 | Yes |
| 320 | PRKDC | "protein kinase, DNA-activated, catalytic subunit [Source:HGNC Symbol;Acc:HGNC:9413]" | 9632 | -0.286 | -0.3121 | Yes |
| 321 | NPR1 | natriuretic peptide receptor 1 [Source:HGNC Symbol;Acc:HGNC:7943] | 9634 | -0.286 | -0.3103 | Yes |
| 322 | IKBKE | inhibitor of nuclear factor kappa B kinase subunit epsilon [Source:HGNC Symbol;Acc:HGNC:14552] | 9646 | -0.287 | -0.3092 | Yes |
| 323 | KALRN | kalirin RhoGEF kinase [Source:HGNC Symbol;Acc:HGNC:4814] | 9649 | -0.287 | -0.3074 | Yes |
| 324 | MARK2 | microtubule affinity regulating kinase 2 [Source:HGNC Symbol;Acc:HGNC:3332] | 9662 | -0.288 | -0.3065 | Yes |
| 325 | PLK3 | polo like kinase 3 [Source:HGNC Symbol;Acc:HGNC:2154] | 9698 | -0.291 | -0.3073 | Yes |
| 326 | MAPK10 | mitogen-activated protein kinase 10 [Source:HGNC Symbol;Acc:HGNC:6872] | 9699 | -0.291 | -0.3053 | Yes |
| 327 | MAP3K9 | mitogen-activated protein kinase kinase kinase 9 [Source:HGNC Symbol;Acc:HGNC:6861] | 9712 | -0.293 | -0.3043 | Yes |
| 328 | LIMK1 | LIM domain kinase 1 [Source:HGNC Symbol;Acc:HGNC:6613] | 9746 | -0.296 | -0.3050 | Yes |
| 329 | CDC7 | cell division cycle 7 [Source:HGNC Symbol;Acc:HGNC:1745] | 9780 | -0.299 | -0.3056 | Yes |
| 330 | ULK1 | unc-51 like autophagy activating kinase 1 [Source:HGNC Symbol;Acc:HGNC:12558] | 9798 | -0.301 | -0.3049 | Yes |
| 331 | LIMK2 | LIM domain kinase 2 [Source:HGNC Symbol;Acc:HGNC:6614] | 9812 | -0.302 | -0.3039 | Yes |
| 332 | CCL2 | C-C motif chemokine ligand 2 [Source:HGNC Symbol;Acc:HGNC:10618] | 9819 | -0.303 | -0.3024 | Yes |
| 333 | STK17B | serine/threonine kinase 17b [Source:HGNC Symbol;Acc:HGNC:11396] | 9825 | -0.303 | -0.3007 | Yes |
| 334 | BCR | BCR activator of RhoGEF and GTPase [Source:HGNC Symbol;Acc:HGNC:1014] | 9830 | -0.304 | -0.2990 | Yes |
| 335 | CDK20 | cyclin dependent kinase 20 [Source:HGNC Symbol;Acc:HGNC:21420] | 9834 | -0.304 | -0.2972 | Yes |
| 336 | STK38 | serine/threonine kinase 38 [Source:HGNC Symbol;Acc:HGNC:17847] | 9912 | -0.313 | -0.3013 | Yes |
| 337 | TRIM24 | tripartite motif containing 24 [Source:HGNC Symbol;Acc:HGNC:11812] | 9927 | -0.314 | -0.3003 | Yes |
| 338 | MAP2K2 | mitogen-activated protein kinase kinase 2 [Source:HGNC Symbol;Acc:HGNC:6842] | 9932 | -0.315 | -0.2985 | Yes |
| 339 | RNASEL | ribonuclease L [Source:HGNC Symbol;Acc:HGNC:10050] | 9965 | -0.317 | -0.2989 | Yes |
| 340 | MOK | MOK protein kinase [Source:HGNC Symbol;Acc:HGNC:9833] | 9984 | -0.319 | -0.2982 | Yes |
| 341 | PAK4 | p21 (RAC1) activated kinase 4 [Source:HGNC Symbol;Acc:HGNC:16059] | 10004 | -0.322 | -0.2975 | Yes |
| 342 | EIF2AK3 | eukaryotic translation initiation factor 2 alpha kinase 3 [Source:HGNC Symbol;Acc:HGNC:3255] | 10025 | -0.324 | -0.2969 | Yes |
| 343 | MAPK4 | mitogen-activated protein kinase 4 [Source:HGNC Symbol;Acc:HGNC:6878] | 10042 | -0.325 | -0.2960 | Yes |
| 344 | FYN | "FYN proto-oncogene, Src family tyrosine kinase [Source:HGNC Symbol;Acc:HGNC:4037]" | 10092 | -0.330 | -0.2977 | Yes |
| 345 | EPHB4 | EPH receptor B4 [Source:HGNC Symbol;Acc:HGNC:3395] | 10097 | -0.330 | -0.2958 | Yes |
| 346 | BAZ1B | bromodomain adjacent to zinc finger domain 1B [Source:HGNC Symbol;Acc:HGNC:961] | 10112 | -0.332 | -0.2947 | Yes |
| 347 | MAP3K3 | mitogen-activated protein kinase kinase kinase 3 [Source:HGNC Symbol;Acc:HGNC:6855] | 10121 | -0.333 | -0.2931 | Yes |
| 348 | MAP4K4 | mitogen-activated protein kinase kinase kinase kinase 4 [Source:HGNC Symbol;Acc:HGNC:6866] | 10134 | -0.334 | -0.2918 | Yes |
| 349 | SIK2 | salt inducible kinase 2 [Source:HGNC Symbol;Acc:HGNC:21680] | 10189 | -0.339 | -0.2939 | Yes |
| 350 | MAP2K1 | mitogen-activated protein kinase kinase 1 [Source:HGNC Symbol;Acc:HGNC:6840] | 10197 | -0.340 | -0.2921 | Yes |
| 351 | EPHA2 | EPH receptor A2 [Source:HGNC Symbol;Acc:HGNC:3386] | 10219 | -0.343 | -0.2915 | Yes |
| 352 | RPS6KB1 | ribosomal protein S6 kinase B1 [Source:HGNC Symbol;Acc:HGNC:10436] | 10224 | -0.343 | -0.2895 | Yes |
| 353 | EFNB3 | ephrin B3 [Source:HGNC Symbol;Acc:HGNC:3228] | 10232 | -0.345 | -0.2877 | Yes |
| 354 | MAK | male germ cell associated kinase [Source:HGNC Symbol;Acc:HGNC:6816] | 10237 | -0.345 | -0.2857 | Yes |
| 355 | GAK | cyclin G associated kinase [Source:HGNC Symbol;Acc:HGNC:4113] | 10301 | -0.350 | -0.2884 | Yes |
| 356 | MAP3K6 | mitogen-activated protein kinase kinase kinase 6 [Source:HGNC Symbol;Acc:HGNC:6858] | 10317 | -0.353 | -0.2872 | Yes |
| 357 | DMPK | DM1 protein kinase [Source:HGNC Symbol;Acc:HGNC:2933] | 10331 | -0.354 | -0.2859 | Yes |
| 358 | TRIO | trio Rho guanine nucleotide exchange factor [Source:HGNC Symbol;Acc:HGNC:12303] | 10362 | -0.357 | -0.2859 | Yes |
| 359 | HIPK1 | homeodomain interacting protein kinase 1 [Source:HGNC Symbol;Acc:HGNC:19006] | 10391 | -0.360 | -0.2857 | Yes |
| 360 | ITPKA | inositol-trisphosphate 3-kinase A [Source:HGNC Symbol;Acc:HGNC:6178] | 10450 | -0.367 | -0.2879 | Yes |
| 361 | INSRR | insulin receptor related receptor [Source:HGNC Symbol;Acc:HGNC:6093] | 10459 | -0.369 | -0.2860 | Yes |
| 362 | PRKACG | protein kinase cAMP-activated catalytic subunit gamma [Source:HGNC Symbol;Acc:HGNC:9382] | 10466 | -0.370 | -0.2840 | Yes |
| 363 | SRPK2 | SRSF protein kinase 2 [Source:HGNC Symbol;Acc:HGNC:11306] | 10522 | -0.376 | -0.2859 | Yes |
| 364 | CCL8 | C-C motif chemokine ligand 8 [Source:HGNC Symbol;Acc:HGNC:10635] | 10529 | -0.376 | -0.2838 | Yes |
| 365 | AMHR2 | anti-Mullerian hormone receptor type 2 [Source:HGNC Symbol;Acc:HGNC:465] | 10571 | -0.381 | -0.2846 | Yes |
| 366 | PRKACA | protein kinase cAMP-activated catalytic subunit alpha [Source:HGNC Symbol;Acc:HGNC:9380] | 10611 | -0.384 | -0.2851 | Yes |
| 367 | MST1R | macrophage stimulating 1 receptor [Source:HGNC Symbol;Acc:HGNC:7381] | 10618 | -0.385 | -0.2830 | Yes |
| 368 | ROCK2 | Rho associated coiled-coil containing protein kinase 2 [Source:HGNC Symbol;Acc:HGNC:10252] | 10648 | -0.388 | -0.2827 | Yes |
| 369 | STK3 | serine/threonine kinase 3 [Source:HGNC Symbol;Acc:HGNC:11406] | 10656 | -0.388 | -0.2806 | Yes |
| 370 | CDK13 | cyclin dependent kinase 13 [Source:HGNC Symbol;Acc:HGNC:1733] | 10675 | -0.390 | -0.2794 | Yes |
| 371 | MAST2 | microtubule associated serine/threonine kinase 2 [Source:HGNC Symbol;Acc:HGNC:19035] | 10703 | -0.394 | -0.2790 | Yes |
| 372 | TAOK2 | TAO kinase 2 [Source:HGNC Symbol;Acc:HGNC:16835] | 10718 | -0.395 | -0.2774 | Yes |
| 373 | TYK2 | tyrosine kinase 2 [Source:HGNC Symbol;Acc:HGNC:12440] | 10756 | -0.399 | -0.2777 | Yes |
| 374 | CAMK1D | calcium/calmodulin dependent protein kinase ID [Source:HGNC Symbol;Acc:HGNC:19341] | 10775 | -0.403 | -0.2764 | Yes |
| 375 | PRKG1 | protein kinase cGMP-dependent 1 [Source:HGNC Symbol;Acc:HGNC:9414] | 10776 | -0.403 | -0.2737 | Yes |
| 376 | WNK1 | WNK lysine deficient protein kinase 1 [Source:HGNC Symbol;Acc:HGNC:14540] | 10777 | -0.403 | -0.2710 | Yes |
| 377 | FLT1 | fms related receptor tyrosine kinase 1 [Source:HGNC Symbol;Acc:HGNC:3763] | 10826 | -0.408 | -0.2721 | Yes |
| 378 | PHKG2 | phosphorylase kinase catalytic subunit gamma 2 [Source:HGNC Symbol;Acc:HGNC:8931] | 10830 | -0.408 | -0.2695 | Yes |
| 379 | IRAK4 | interleukin 1 receptor associated kinase 4 [Source:HGNC Symbol;Acc:HGNC:17967] | 10874 | -0.413 | -0.2702 | Yes |
| 380 | JAK3 | Janus kinase 3 [Source:HGNC Symbol;Acc:HGNC:6193] | 10908 | -0.418 | -0.2700 | Yes |
| 381 | MAP3K5 | mitogen-activated protein kinase kinase kinase 5 [Source:HGNC Symbol;Acc:HGNC:6857] | 10923 | -0.421 | -0.2683 | Yes |
| 382 | ALPK3 | alpha kinase 3 [Source:HGNC Symbol;Acc:HGNC:17574] | 10973 | -0.427 | -0.2694 | Yes |
| 383 | CDK5R1 | cyclin dependent kinase 5 regulatory subunit 1 [Source:HGNC Symbol;Acc:HGNC:1775] | 10985 | -0.428 | -0.2674 | Yes |
| 384 | ACVR2B | activin A receptor type 2B [Source:HGNC Symbol;Acc:HGNC:174] | 11011 | -0.431 | -0.2665 | Yes |
| 385 | MARK1 | microtubule affinity regulating kinase 1 [Source:HGNC Symbol;Acc:HGNC:6896] | 11031 | -0.434 | -0.2651 | Yes |
| 386 | MARK3 | microtubule affinity regulating kinase 3 [Source:HGNC Symbol;Acc:HGNC:6897] | 11063 | -0.440 | -0.2646 | Yes |
| 387 | CDK2 | cyclin dependent kinase 2 [Source:HGNC Symbol;Acc:HGNC:1771] | 11069 | -0.441 | -0.2620 | Yes |
| 388 | CDK9 | cyclin dependent kinase 9 [Source:HGNC Symbol;Acc:HGNC:1780] | 11071 | -0.441 | -0.2591 | Yes |
| 389 | STK4 | serine/threonine kinase 4 [Source:HGNC Symbol;Acc:HGNC:11408] | 11074 | -0.441 | -0.2563 | Yes |
| 390 | TRPM6 | transient receptor potential cation channel subfamily M member 6 [Source:HGNC Symbol;Acc:HGNC:17995] | 11080 | -0.442 | -0.2537 | Yes |
| 391 | CDKL1 | cyclin dependent kinase like 1 [Source:HGNC Symbol;Acc:HGNC:1781] | 11109 | -0.444 | -0.2529 | Yes |
| 392 | STK26 | serine/threonine kinase 26 [Source:HGNC Symbol;Acc:HGNC:18174] | 11152 | -0.451 | -0.2533 | Yes |
| 393 | GSK3B | glycogen synthase kinase 3 beta [Source:HGNC Symbol;Acc:HGNC:4617] | 11157 | -0.451 | -0.2505 | Yes |
| 394 | BMPR2 | bone morphogenetic protein receptor type 2 [Source:HGNC Symbol;Acc:HGNC:1078] | 11174 | -0.454 | -0.2488 | Yes |
| 395 | LTK | leukocyte receptor tyrosine kinase [Source:HGNC Symbol;Acc:HGNC:6721] | 11210 | -0.458 | -0.2485 | Yes |
| 396 | ATR | ATR serine/threonine kinase [Source:HGNC Symbol;Acc:HGNC:882] | 11231 | -0.461 | -0.2470 | Yes |
| 397 | ALK | ALK receptor tyrosine kinase [Source:HGNC Symbol;Acc:HGNC:427] | 11262 | -0.466 | -0.2462 | Yes |
| 398 | CSNK1A1 | casein kinase 1 alpha 1 [Source:HGNC Symbol;Acc:HGNC:2451] | 11310 | -0.472 | -0.2468 | Yes |
| 399 | TBK1 | TANK binding kinase 1 [Source:HGNC Symbol;Acc:HGNC:11584] | 11326 | -0.474 | -0.2448 | Yes |
| 400 | CCNK | cyclin K [Source:HGNC Symbol;Acc:HGNC:1596] | 11330 | -0.475 | -0.2419 | Yes |
| 401 | MAP3K12 | mitogen-activated protein kinase kinase kinase 12 [Source:HGNC Symbol;Acc:HGNC:6851] | 11344 | -0.478 | -0.2397 | Yes |
| 402 | CLK2 | CDC like kinase 2 [Source:HGNC Symbol;Acc:HGNC:2069] | 11368 | -0.482 | -0.2383 | Yes |
| 403 | PDK4 | pyruvate dehydrogenase kinase 4 [Source:HGNC Symbol;Acc:HGNC:8812] | 11384 | -0.483 | -0.2362 | Yes |
| 404 | PKMYT1 | "protein kinase, membrane associated tyrosine/threonine 1 [Source:HGNC Symbol;Acc:HGNC:29650]" | 11403 | -0.487 | -0.2344 | Yes |
| 405 | STK24 | serine/threonine kinase 24 [Source:HGNC Symbol;Acc:HGNC:11403] | 11409 | -0.488 | -0.2315 | Yes |
| 406 | CRIM1 | cysteine rich transmembrane BMP regulator 1 [Source:HGNC Symbol;Acc:HGNC:2359] | 11425 | -0.492 | -0.2293 | Yes |
| 407 | STK11 | serine/threonine kinase 11 [Source:HGNC Symbol;Acc:HGNC:11389] | 11451 | -0.496 | -0.2280 | Yes |
| 408 | KDR | kinase insert domain receptor [Source:HGNC Symbol;Acc:HGNC:6307] | 11454 | -0.496 | -0.2248 | Yes |
| 409 | CCL3 | C-C motif chemokine ligand 3 [Source:HGNC Symbol;Acc:HGNC:10627] | 11465 | -0.497 | -0.2222 | Yes |
| 410 | MAP3K20 | mitogen-activated protein kinase kinase kinase 20 [Source:HGNC Symbol;Acc:HGNC:17797] | 11475 | -0.498 | -0.2196 | Yes |
| 411 | CSNK1E | casein kinase 1 epsilon [Source:HGNC Symbol;Acc:HGNC:2453] | 11491 | -0.500 | -0.2174 | Yes |
| 412 | PRKAA1 | protein kinase AMP-activated catalytic subunit alpha 1 [Source:HGNC Symbol;Acc:HGNC:9376] | 11499 | -0.501 | -0.2146 | Yes |
| 413 | MAPK11 | mitogen-activated protein kinase 11 [Source:HGNC Symbol;Acc:HGNC:6873] | 11501 | -0.501 | -0.2113 | Yes |
| 414 | PTK2B | protein tyrosine kinase 2 beta [Source:HGNC Symbol;Acc:HGNC:9612] | 11618 | -0.520 | -0.2171 | Yes |
| 415 | CHUK | component of inhibitor of nuclear factor kappa B kinase complex [Source:HGNC Symbol;Acc:HGNC:1974] | 11624 | -0.521 | -0.2140 | Yes |
| 416 | PRKX | protein kinase X-linked [Source:HGNC Symbol;Acc:HGNC:9441] | 11634 | -0.522 | -0.2112 | Yes |
| 417 | AATK | apoptosis associated tyrosine kinase [Source:HGNC Symbol;Acc:HGNC:21] | 11720 | -0.536 | -0.2144 | Yes |
| 418 | MAP3K10 | mitogen-activated protein kinase kinase kinase 10 [Source:HGNC Symbol;Acc:HGNC:6849] | 11753 | -0.541 | -0.2133 | Yes |
| 419 | LATS1 | large tumor suppressor kinase 1 [Source:HGNC Symbol;Acc:HGNC:6514] | 11760 | -0.542 | -0.2101 | Yes |
| 420 | COQ8B | coenzyme Q8B [Source:HGNC Symbol;Acc:HGNC:19041] | 11763 | -0.544 | -0.2066 | Yes |
| 421 | LMTK2 | lemur tyrosine kinase 2 [Source:HGNC Symbol;Acc:HGNC:17880] | 11764 | -0.544 | -0.2029 | Yes |
| 422 | TOP1 | DNA topoisomerase I [Source:HGNC Symbol;Acc:HGNC:11986] | 11828 | -0.555 | -0.2043 | Yes |
| 423 | PDPK1 | 3-phosphoinositide dependent protein kinase 1 [Source:HGNC Symbol;Acc:HGNC:8816] | 11839 | -0.557 | -0.2013 | Yes |
| 424 | AAK1 | AP2 associated kinase 1 [Source:HGNC Symbol;Acc:HGNC:19679] | 11842 | -0.558 | -0.1977 | Yes |
| 425 | EPHA4 | EPH receptor A4 [Source:HGNC Symbol;Acc:HGNC:3388] | 11863 | -0.563 | -0.1955 | Yes |
| 426 | SRPK1 | SRSF protein kinase 1 [Source:HGNC Symbol;Acc:HGNC:11305] | 11889 | -0.569 | -0.1936 | Yes |
| 427 | CSNK2A1 | casein kinase 2 alpha 1 [Source:HGNC Symbol;Acc:HGNC:2457] | 11895 | -0.570 | -0.1902 | Yes |
| 428 | TRIB1 | tribbles pseudokinase 1 [Source:HGNC Symbol;Acc:HGNC:16891] | 11924 | -0.577 | -0.1885 | Yes |
| 429 | CDK10 | cyclin dependent kinase 10 [Source:HGNC Symbol;Acc:HGNC:1770] | 11930 | -0.579 | -0.1850 | Yes |
| 430 | CDK11B | cyclin dependent kinase 11B [Source:HGNC Symbol;Acc:HGNC:1729] | 11952 | -0.584 | -0.1828 | Yes |
| 431 | RIPK1 | receptor interacting serine/threonine kinase 1 [Source:HGNC Symbol;Acc:HGNC:10019] | 11953 | -0.584 | -0.1788 | Yes |
| 432 | STYK1 | serine/threonine/tyrosine kinase 1 [Source:HGNC Symbol;Acc:HGNC:18889] | 11956 | -0.584 | -0.1750 | Yes |
| 433 | MAP3K8 | mitogen-activated protein kinase kinase kinase 8 [Source:HGNC Symbol;Acc:HGNC:6860] | 11975 | -0.589 | -0.1725 | Yes |
| 434 | MAP2K3 | mitogen-activated protein kinase kinase 3 [Source:HGNC Symbol;Acc:HGNC:6843] | 12004 | -0.593 | -0.1707 | Yes |
| 435 | ROCK1 | Rho associated coiled-coil containing protein kinase 1 [Source:HGNC Symbol;Acc:HGNC:10251] | 12046 | -0.605 | -0.1699 | Yes |
| 436 | MTOR | mechanistic target of rapamycin kinase [Source:HGNC Symbol;Acc:HGNC:3942] | 12047 | -0.605 | -0.1658 | Yes |
| 437 | TTBK2 | tau tubulin kinase 2 [Source:HGNC Symbol;Acc:HGNC:19141] | 12083 | -0.615 | -0.1645 | Yes |
| 438 | MAPKAPK2 | MAPK activated protein kinase 2 [Source:HGNC Symbol;Acc:HGNC:6887] | 12094 | -0.619 | -0.1611 | Yes |
| 439 | FER | FER tyrosine kinase [Source:HGNC Symbol;Acc:HGNC:3655] | 12101 | -0.620 | -0.1574 | Yes |
| 440 | EGFR | epidermal growth factor receptor [Source:HGNC Symbol;Acc:HGNC:3236] | 12109 | -0.622 | -0.1538 | Yes |
| 441 | NTRK3 | neurotrophic receptor tyrosine kinase 3 [Source:HGNC Symbol;Acc:HGNC:8033] | 12157 | -0.632 | -0.1533 | Yes |
| 442 | DAPK3 | death associated protein kinase 3 [Source:HGNC Symbol;Acc:HGNC:2676] | 12180 | -0.639 | -0.1507 | Yes |
| 443 | MAP2K7 | mitogen-activated protein kinase kinase 7 [Source:HGNC Symbol;Acc:HGNC:6847] | 12209 | -0.649 | -0.1486 | Yes |
| 444 | MKNK2 | MAPK interacting serine/threonine kinase 2 [Source:HGNC Symbol;Acc:HGNC:7111] | 12236 | -0.655 | -0.1463 | Yes |
| 445 | PKN2 | protein kinase N2 [Source:HGNC Symbol;Acc:HGNC:9406] | 12237 | -0.655 | -0.1418 | Yes |
| 446 | ACVRL1 | activin A receptor like type 1 [Source:HGNC Symbol;Acc:HGNC:175] | 12242 | -0.656 | -0.1377 | Yes |
| 447 | SMG1 | SMG1 nonsense mediated mRNA decay associated PI3K related kinase [Source:HGNC Symbol;Acc:HGNC:30045] | 12268 | -0.668 | -0.1352 | Yes |
| 448 | CDK3 | cyclin dependent kinase 3 [Source:HGNC Symbol;Acc:HGNC:1772] | 12337 | -0.687 | -0.1360 | Yes |
| 449 | ABL2 | "ABL proto-oncogene 2, non-receptor tyrosine kinase [Source:HGNC Symbol;Acc:HGNC:77]" | 12342 | -0.688 | -0.1317 | Yes |
| 450 | PIKFYVE | "phosphoinositide kinase, FYVE-type zinc finger containing [Source:HGNC Symbol;Acc:HGNC:23785]" | 12347 | -0.690 | -0.1273 | Yes |
| 451 | CLK1 | CDC like kinase 1 [Source:HGNC Symbol;Acc:HGNC:2068] | 12354 | -0.693 | -0.1231 | Yes |
| 452 | SGK1 | serum/glucocorticoid regulated kinase 1 [Source:HGNC Symbol;Acc:HGNC:10810] | 12357 | -0.694 | -0.1186 | Yes |
| 453 | DYRK3 | dual specificity tyrosine phosphorylation regulated kinase 3 [Source:HGNC Symbol;Acc:HGNC:3094] | 12379 | -0.702 | -0.1155 | Yes |
| 454 | IRAK3 | interleukin 1 receptor associated kinase 3 [Source:HGNC Symbol;Acc:HGNC:17020] | 12383 | -0.703 | -0.1110 | Yes |
| 455 | GSK3A | glycogen synthase kinase 3 alpha [Source:HGNC Symbol;Acc:HGNC:4616] | 12384 | -0.703 | -0.1063 | Yes |
| 456 | FLT4 | fms related receptor tyrosine kinase 4 [Source:HGNC Symbol;Acc:HGNC:3767] | 12396 | -0.705 | -0.1024 | Yes |
| 457 | HIPK3 | homeodomain interacting protein kinase 3 [Source:HGNC Symbol;Acc:HGNC:4915] | 12404 | -0.707 | -0.0982 | Yes |
| 458 | SCYL2 | SCY1 like pseudokinase 2 [Source:HGNC Symbol;Acc:HGNC:19286] | 12439 | -0.720 | -0.0960 | Yes |
| 459 | PRKCI | protein kinase C iota [Source:HGNC Symbol;Acc:HGNC:9404] | 12444 | -0.723 | -0.0915 | Yes |
| 460 | YES1 | "YES proto-oncogene 1, Src family tyrosine kinase [Source:HGNC Symbol;Acc:HGNC:12841]" | 12450 | -0.725 | -0.0870 | Yes |
| 461 | ALPK1 | alpha kinase 1 [Source:HGNC Symbol;Acc:HGNC:20917] | 12481 | -0.741 | -0.0844 | Yes |
| 462 | PIM1 | "Pim-1 proto-oncogene, serine/threonine kinase [Source:HGNC Symbol;Acc:HGNC:8986]" | 12500 | -0.748 | -0.0808 | Yes |
| 463 | PRKY | protein kinase Y-linked (pseudogene) [Source:HGNC Symbol;Acc:HGNC:9444] | 12560 | -0.777 | -0.0803 | Yes |
| 464 | AKAP13 | A-kinase anchoring protein 13 [Source:HGNC Symbol;Acc:HGNC:371] | 12572 | -0.782 | -0.0758 | Yes |
| 465 | BRD4 | bromodomain containing 4 [Source:HGNC Symbol;Acc:HGNC:13575] | 12606 | -0.800 | -0.0731 | Yes |
| 466 | IGF1R | insulin like growth factor 1 receptor [Source:HGNC Symbol;Acc:HGNC:5465] | 12689 | -0.855 | -0.0739 | Yes |
| 467 | SNRK | SNF related kinase [Source:HGNC Symbol;Acc:HGNC:30598] | 12700 | -0.864 | -0.0689 | Yes |
| 468 | BRD2 | bromodomain containing 2 [Source:HGNC Symbol;Acc:HGNC:1103] | 12709 | -0.871 | -0.0636 | Yes |
| 469 | CDKN1A | cyclin dependent kinase inhibitor 1A [Source:HGNC Symbol;Acc:HGNC:1784] | 12727 | -0.890 | -0.0590 | Yes |
| 470 | CSNK1G2 | casein kinase 1 gamma 2 [Source:HGNC Symbol;Acc:HGNC:2455] | 12729 | -0.890 | -0.0530 | Yes |
| 471 | PRKAB2 | protein kinase AMP-activated non-catalytic subunit beta 2 [Source:HGNC Symbol;Acc:HGNC:9379] | 12731 | -0.893 | -0.0471 | Yes |
| 472 | TAOK1 | TAO kinase 1 [Source:HGNC Symbol;Acc:HGNC:29259] | 12753 | -0.918 | -0.0426 | Yes |
| 473 | DDR2 | discoidin domain receptor tyrosine kinase 2 [Source:HGNC Symbol;Acc:HGNC:2731] | 12819 | -1.003 | -0.0410 | Yes |
| 474 | HIPK2 | homeodomain interacting protein kinase 2 [Source:HGNC Symbol;Acc:HGNC:14402] | 12842 | -1.045 | -0.0357 | Yes |
| 475 | INSR | insulin receptor [Source:HGNC Symbol;Acc:HGNC:6091] | 12856 | -1.071 | -0.0295 | Yes |
| 476 | SIK1 | salt inducible kinase 1 [Source:HGNC Symbol;Acc:HGNC:11142] | 12863 | -1.104 | -0.0225 | Yes |
| 477 | GRK1 | G protein-coupled receptor kinase 1 [Source:HGNC Symbol;Acc:HGNC:10013] | 12874 | -1.139 | -0.0156 | Yes |
| 478 | TAF1 | TATA-box binding protein associated factor 1 [Source:HGNC Symbol;Acc:HGNC:11535] | 12881 | -1.192 | -0.0080 | Yes |
| 479 | AKT2 | AKT serine/threonine kinase 2 [Source:HGNC Symbol;Acc:HGNC:392] | 12896 | -1.404 | 0.0003 | Yes |
Table: GSEA details [plain text format]

  

Fig 2: GOMF\_PROTEIN\_KINASE\_ACTIVITY      
 Blue-Pink O' Gram in the Space of the Analyzed GeneSet

  

Fig 3: GOMF\_PROTEIN\_KINASE\_ACTIVITY: Random ES distribution      
 Gene set null distribution of ES for **GOMF\_PROTEIN\_KINASE\_ACTIVITY**
